# P-2100. Comparison of Fosfomycin MIC Values between Parent and Inner Colonies (IC) of *Escherichia coli* Arising During Disk Diffusion (DD) Testing

**DOI:** 10.1093/ofid/ofae631.2256

**Published:** 2025-01-29

**Authors:** Khadijah Malik, Jenna Salay, Morgan L Bixby, Morgan L Bixby, Lindsey Collins, Tiffany Chang, Elizabeth B Hirsch

**Affiliations:** University of Minnesota College of Pharmacy, Minneapolis, Minnesota; University of Minnesota College of Pharmacy, Minneapolis, Minnesota; Univeristy of Minnesota, Saint Paul, Minnesota; Univeristy of Minnesota, Saint Paul, Minnesota; University of Minnesota College of Pharmacy, Minneapolis, Minnesota; University of Minnesota, Twin Cities, Minneapolis, Minnesota; University of Minnesota College of Pharmacy, Minneapolis, Minnesota

## Abstract

**Background:**

Recent studies have documented uropathogenic *E. coli* displaying the presence of IC during fosfomycin DD testing. These IC often demonstrate increased minimal inhibitory concentrations (MIC) when compared to their parent isolates. Investigating the clinical relevance of these IC and the extent of their resistance is a first step in determining whether they are associated with fosfomycin failure.

**Figure:**
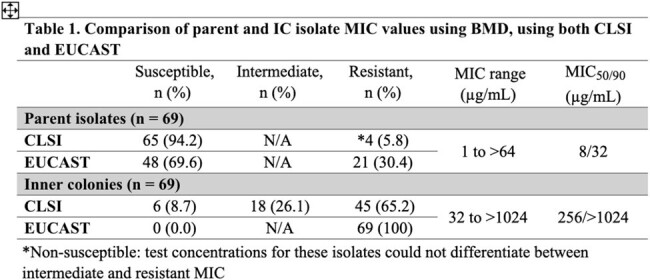

**Methods:**

*E. coli* isolates (n = 69) collected from the University of Minnesota Medical Center (UMMC) that produced ≥ 3 discrete IC during at least one DD test were included. Parent and IC (n = 69) isolates underwent broth microdilution (BMD) testing using fosfomycin concentrations of 0.5-64 µg/mL for parent isolates and 8-1024 µg/mL for IC with incubation at 37°C for 16-20 hours. All susceptibility testing was supplemented with 25 µg/mL glucose-6-phosphate and conducted in duplicate on separate days. *E. faecalis* ATCC 29212 and *E. coli* ATCC 25922 were included as quality control strains. The results were evaluated using both CLSI (susceptible [S]: 64 µg/mL) and EUCAST (S: 8 µg/mL) breakpoints. Categorical agreement between the two guidelines were calculated.

**Figure:**
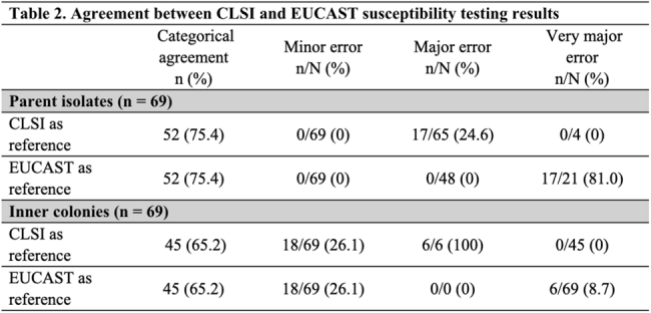

**Results:**

*E. coli* parent isolates had an MIC range of 1 to >64 µg/mL with a MIC_50/90_ of 8/32 µg/mL (Table 1). The IC isolates had significantly higher MIC ranges (< 8 and >1024 µg/mL) and MIC_50/90_ values of 256/ >1024 µg/mL. The parent isolates had 94.2% (n = 65) and 69.6% (n = 48) susceptibility per CLSI and EUCAST breakpoints, respectively. Using the more stringent EUCAST breakpoints, all IC isolates were resistant whereas 65.2% (n = 45) were considered resistant per CLSI breakpoints. Categorical agreement among the parent collection was 75.4% and 65.2% in the IC collection (Table 2).

**Conclusion:**

Our data indicate IC arising from *E. coli* during fosfomycin DD testing are notably more resistant than their parent isolates, with an MIC_50_ increase of five, 2-fold dilutions. Future directions for this work include expanding the isolate collection and examining the fitness of these IC in comparison to their parents. Improving the understanding of the frequency, resistance, and fitness of IC arising from fosfomycin DD testing will help determine an optimal interpretation of IC, which may in turn improve patient outcomes.

**Disclosures:**

Elizabeth B. Hirsch, PharmD, FCCP, FIDSA, GSK: Advisor/Consultant|GSK: Honoraria

